# Spectrum of *PEX1* and *PEX6* variants in Heimler syndrome

**DOI:** 10.1038/ejhg.2016.62

**Published:** 2016-06-15

**Authors:** Claire E L Smith, James A Poulter, Alex V Levin, Jenina E Capasso, Susan Price, Tamar Ben-Yosef, Reuven Sharony, William G Newman, Roger C Shore, Steven J Brookes, Alan J Mighell, Chris F Inglehearn

**Affiliations:** 1 Leeds Institute of Biomedical and Clinical Sciences, St. James's University Hospital, University of Leeds, Leeds, UK; 2 Sidney Kimmel Medical College at Thomas Jefferson University, Philadelphia, PA, USA; 3 Children's Hospital of the King's Daughters, Norfolk, VA, USA; 4 Pediatric Ophthalmology and Ocular Genetics, Philadelphia, PA, USA; 5 Department of Clinical Genetics, Northampton General Hospital, NHS Trust, Northampton, UK; 6 Rappaport Faculty of Medicine, Technion, Haifa, Israel; 7 The Genetic Institute and Obstetrics and Gynaecology Department, Meir Medical Center, Kfar Saba, Israel; 8 Manchester Centre for Genomic Medicine, St. Mary's Hospital, Manchester Academic Health Sciences Centre, Manchester, UK; 9 Manchester Centre for Genomic Medicine, Institute of Human Development, University of Manchester, Manchester, UK; 10 School of Dentistry, Department of Oral Biology, St. James's University Hospital, University of Leeds, Leeds, UK; 11 Department of Oral Medicine, School of Dentistry, University of Leeds, Leeds, UK

## Abstract

Heimler syndrome (HS) consists of recessively inherited sensorineural hearing loss, amelogenesis imperfecta (AI) and nail abnormalities, with or without visual defects. Recently HS was shown to result from hypomorphic mutations in *PEX1* or *PEX6*, both previously implicated in Zellweger Syndrome Spectrum Disorders (ZSSD). ZSSD are a group of conditions consisting of craniofacial and neurological abnormalities, sensory defects and multi-organ dysfunction. The finding of HS-causing mutations in *PEX1* and *PEX6* shows that HS represents the mild end of the ZSSD spectrum, though these conditions were previously thought to be distinct nosological entities. Here, we present six further HS families, five with *PEX6* variants and one with *PEX1* variants, and show the patterns of Pex1, Pex14 and Pex6 immunoreactivity in the mouse retina. While Ratbi *et al.* found more HS-causing mutations in *PEX1* than in *PEX6*, as is the case for ZSSD, in this cohort *PEX6* variants predominate, suggesting both genes play a significant role in HS. The *PEX6* variant c.1802G>A, p.(R601Q), reported previously in compound heterozygous state in one HS and three ZSSD cases, was found in compound heterozygous state in three HS families. Haplotype analysis suggests a common founder variant. All families segregated at least one missense variant, consistent with the hypothesis that HS results from genotypes including milder hypomorphic alleles. The clinical overlap of HS with the more common Usher syndrome and lack of peroxisomal abnormalities on plasma screening suggest that HS may be under-diagnosed. Recognition of AI is key to the accurate diagnosis of HS.

## Introduction

Heimler syndrome (HS; #234580, #616617) was first described as a combination of sensorineural hearing loss, amelogenesis imperfecta (AI) and nail abnormalities in two siblings.^1^ Subsequently, three further families were reported, detailing five patients with similar phenotypes, all with family histories consistent with recessive inheritance.^2, 3, 4^ Additionally, one of the original cases developed macular dystrophy at 29 years of age, leading to suspicion that HS may also encompass eye disease in either its clinical course or phenotypic spectrum.^5^

A recent study described mutations in *Peroxisomal Biogenesis Factor 1* (*PEX1*; MIM*602136) and *Peroxisomal Biogenesis Factor 6* (*PEX6*; MIM*601498) as the cause of HS in a patient cohort.^6^ Four families, including the index cases, had biallelic mutations in *PEX1,* while two families segregated biallelic mutations in *PEX6*.^6^ These genes, together with 12 other *PEX* genes, are implicated in peroxisome biogenesis disorders (PBDs; MIM Phenotypic series PS214100), which are characterised by a wide range of phenotypes, including craniofacial dysmorphism, neurological abnormalities, sensory defects and liver, kidney and bone abnormalities.^7^ The PBDs include the Zellweger syndrome spectrum disorders (ZSSD): Zellweger syndrome (ZS), neonatal adrenoleukodystrophy (NALD) and infantile Refsum disease (IRD), which represent overlapping clinical phenotypes that vary in severity, with ZS being the most severe. While ZS patients often present with serious disease at birth and live only a few weeks or months, patients with IRD and NALD generally present later in childhood, primarily with sensorineural hearing loss and retinal dystrophy, but also with multiple organ dysfunction and psychomotor impairments.^7, 8^ By comparison, HS patients therefore represent the mildest phenotypic subgroup of the ZSSD.^6^

Mutations in *PEX1* and *PEX6* are the most common causes of ZSSD and result in impaired peroxisomal function.^9^ Peroxisomes are ubiquitous cellular organelles that perform numerous diverse vital functions, including β-oxidation of very long-chain fatty acids, the synthesis of myelin precursors and detoxification of hydrogen peroxide. Defective peroxisomal function can result in changes in neuronal migration, proliferation, differentiation and survival.^10, 11, 12^

PEX1 and PEX6 are type 2 AAA+ ATPases that form a heterohexameric protein complex *in vivo*.^13^ The complex is part of the mechanism that shuttles the peroxisome-targeting signal receptor protein PEX5 back to the cytosol after release of its protein cargo within the peroxisomal lumen. PEX1 and PEX6 provide the energy required to remove PEX5 from the luminal membrane and their function is dependent upon interaction with the membrane protein PEX26.^14^ Peroxisomes are formed primarily by fission following import of newly synthesised peroxisomal proteins from the cytoplasm into the peroxisome. Mutations in the genes encoding these proteins therefore give rise to a peroxisomal protein import defect, which in turn leads to a deficiency of peroxisomal biogenesis.

The phenotype variation seen in ZSSD is related to the severity of the underlying *PEX* gene mutations. Biallelic loss of function or null alleles, caused by frameshift and nonsense mutations, often lead to a total absence of peroxisomes, resulting in ZS, while genotypes that include an allele with a minor import defect, caused by a missense mutation, may lead to NALD or IRD.^15, 16, 17^ Similarly HS was also shown to result from hypomorphic mutations.^6^ Functional complementation studies of *PEX1* and *PEX6* variants in peroxisome-deficient cells revealed that at least one of the HS alleles in each patient retained significant activity.^6^ Therefore the aetiological basis of the HS phenotype is believed to be a mild peroxisomal protein import defect that is the result either of biallelic hypomorphic alleles or of compound heterozygosity for a genotype involving at least one hypomorphic allele.^6^

Here we present six additional families in whom affected individuals were diagnosed with sensorineural hearing loss, enamel defects and retinal dystrophy. Whole-exome sequencing revealed biallelic *PEX1* or *PEX6* variants in each family. We also present patterns of immunoreactivity for the peroxisomal proteins Pex1, Pex6 and Pex14 in adult mouse retina.

## Subjects And Methods

### Patients

HS patients and relatives from six unrelated families were recruited after obtaining informed consent, in accordance with the principles outlined in the declaration of Helsinki, with local ethical approval. Genomic DNA was obtained from venous blood samples using a salt-based extraction protocol or from Saliva using Oragene DNA Sample Collection kits (DNA Genotek, Ottawa, ON, Canada) as detailed in the manufacturer's instructions.

### Whole-exome sequencing and analysis

Three micrograms of genomic DNA from single or multiple individuals from each family (marked with * on pedigrees, Supplementary Figure S1) was subjected to whole-exome sequencing using the SureSelect All Exon v4 or v5 XT reagent (Agilent Technologies, Santa Clara, CA, USA). Sequencing was performed on an Illumina Hi-Seq 2500 sequencing platform (Illumina, San Diego, CA, USA), using a 100 bp paired-end protocol. Fastq files were aligned to the human reference genome (GRCh37) using Novoalign software (Novocraft Technologies, Selangor, Malaysia). The resulting alignment was processed in the SAM/BAM format using the SAMtools, Picard (http://picard.sourceforge.net) and GATK programs to correct alignments around indel sites and mark potential PCR duplicates.^18, 19^

Indel and single-nucleotide variants were called in the VCF format using the Unified Genotyper function of the GATK program. Using the dbSNP database at NCBI, any variants present in dbSNP142 with a minor allele frequency (MAF) ≥1% were then excluded and the remaining variants were annotated using in-house software freely available at http://sourceforge.net/projects/vcfhacks/.

### PCR and Sanger sequencing

Variants were confirmed and segregation was tested in all available family members. Primer sequences can be found in Supplementary Table S1. PCR mastermix HotShot Diamond (Clent Life Science, Stourbridge, UK) was used to amplify sequences. Sanger sequencing was performed using the BigDye Terminator v3.1 kit (Life Technologies, Carlsbad, CA, USA) according to the manufacturer's instructions and resolved on an ABI3130xl sequencer (Life Technologies). Results were analysed using SeqScape v2.5 (Life Technologies).

All variants confirmed by Sanger sequencing and reported in this study were submitted to ClinVar (http://www.ncbi.nlm.nih.gov/clinvar/ submission references SCV000264800- SCV000264808 inclusive).

### Microsatellite marker genotyping

Genotyping of genomic DNA was carried out using fluorescently labelled primers (Sigma, St Louis, MO, USA). Amplified DNA was diluted between 5 and 20 × with Hi-Di Formamide (Applied Biosystems, Foster City, CA, USA) and 1 μl of the dilution added to 8 μl Hi-Di Formamide and 1 μl 500 ROX size standard (Applied Biosystems). Fragments were resolved on an ABI3130xl sequencer using a 36-cm array, POP7 polymer and 3730 buffer with the FragmentAnalysis36_pop7_1 module (Applied Biosystems). Resulting data were analysed on GeneMapper v4.0 (Applied Biosystems).

### Immunohistochemistry

Eye sections were obtained by dissection from killed adult C57Bl/6 mice. After fixation in neutral-buffered formalin for 24 h, eyes were embedded in paraffin wax and sectioned at a thickness of 5 μm. Sections were mounted on SuperFrost Plus slides (Menzel-Glaser, Braunschweig, Germany). Immunohistochemistry was carried out by microwave treatment with 10 mm citrate buffer pH 6.0. Blocking of endogenous peroxidases was achieved by incubating slides with 0.3% hydrogen peroxide in methanol for 10 min. The primary antibodies and the dilutions used were rabbit anti-human PEX14 polyclonal (10594-1-AP; Proteintech, Chicago, IL, USA) at 1:750, rabbit anti-human PEX1 polyclonal antibody (13669-1-AP; Proteintech) at 1:450 and goat anti-rat Pex6 polyclonal antibody (ab175064; Abcam, Cambridge, UK) at 1:50. The anti-PEX14, anti-PEX1 and anti-Pex6 antibodies had been raised against human and rat antigens, respectively, but all were predicted to crossreact with the corresponding murine antigen. The secondary antibody used for sections treated with the anti-PEX14 or the anti-PEX1 antibodies was the labelled polymer-HRP anti-rabbit reagent from the EnVision+ System-HRP (DAB), for use with rabbit primary antibodies (Dako, Ely, UK) and was used as described in the manufacturer's instructions. Slides were washed twice with Tris-buffered saline pH 7.5 with 0.0125% Tween 20 added and once without after each reagent. Staining was achieved with DAB+ reagent from the EnVision+ System-HRP (DAB) for use with rabbit primary antibodies (Dako) as described in the manufacturer's instructions. Counter staining was with haematoxylin (Solmedia, Shrewsbury, UK). Use of the anti-Pex6 antibody raised in goat required some modification to this standard method. Avidin and biotin blocking (Vector Laboratories, Burlingame, CA, USA) was used prior to primary antibody application for the sections treated with the antibody raised in goat. In addition, the secondary antibody and dilution used for these sections was rabbit polyclonal anti-goat immunoglobulins/biotinylated (E0466; Dako) at 1:200. These sections were then treated with the Vectastain Elite ABC Kit (Vector Laboratories) prior to staining with DAB reagent as described above.

## Results

### Whole-exome sequencing

**Figure 1:**
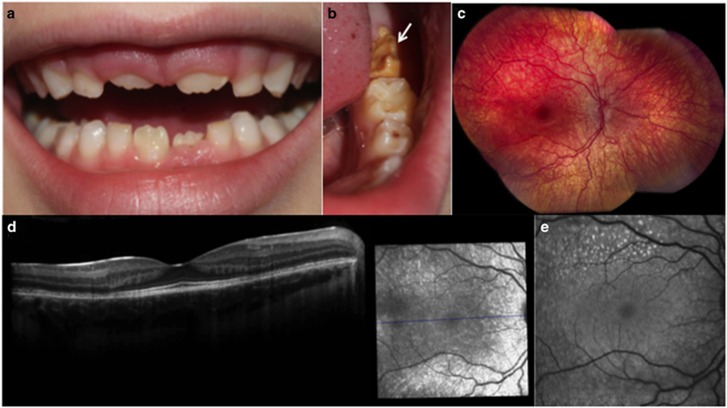
Clinical detail of the phenotype of individual II:1 from Family 3. (**a** and **b**) AI affecting the primary and secondary dentitions with a generalised reduced enamel volume (hypoplasia) and variable hypomineralisation, which is a feature particularly evident in the lower left permanent first molar tooth (white arrow). (**c–e**) The figures detail the phenotype of the right eye. (**c**) Fundus image showing pigmentary maculopathy and mild retina vascular attenuation. (**d**) Optical coherence tomography showing depletion of photoreceptors in the perifovea and disruption of the outer nuclear layer. (**e**) Fundus autofluorescence showing hyperfluorescence at the perifovea.

Six unrelated families segregating autosomal recessive syndromes with phenotypes overlapping HS were recruited to the study. The majority (cases in Families 1, 3, 4, 5 and 6) had been clinically diagnosed with a combination of Usher syndrome (MIM Phenotypic series PS276900) and AI, while only the proband in Family 2 had been diagnosed with HS (Supplementary Figure S2). The phenotype of II:2 of Family 4 has been reported previously.^20^ Affected individuals presented with sensorineural hearing loss, retinal dystrophy and enamel hypoplasia (Figure 1 and Supplementary Table S2). In addition, II:1 of Family 6 presented with schizophrenia, mild learning disability and skin abnormalities over his hands and lower legs (Supplementary Figure S3). Families were of US (Families 1, 2 and 3), UK (Family 4), Israeli (Family 5) and Chinese (Family 6) origins and none reported consanguinity.

Following post-processing and duplicate removal, mean depth of coverage for targeted exons ranged from 37.8 to 89.8 reads. Bases covered by at least five reads ranged from a minimum of 98.5% to a maximum of 99.4% for each of the eight exomes examined. Further alignment statistics can be found in Supplementary Table S3.

**Figure 2:**
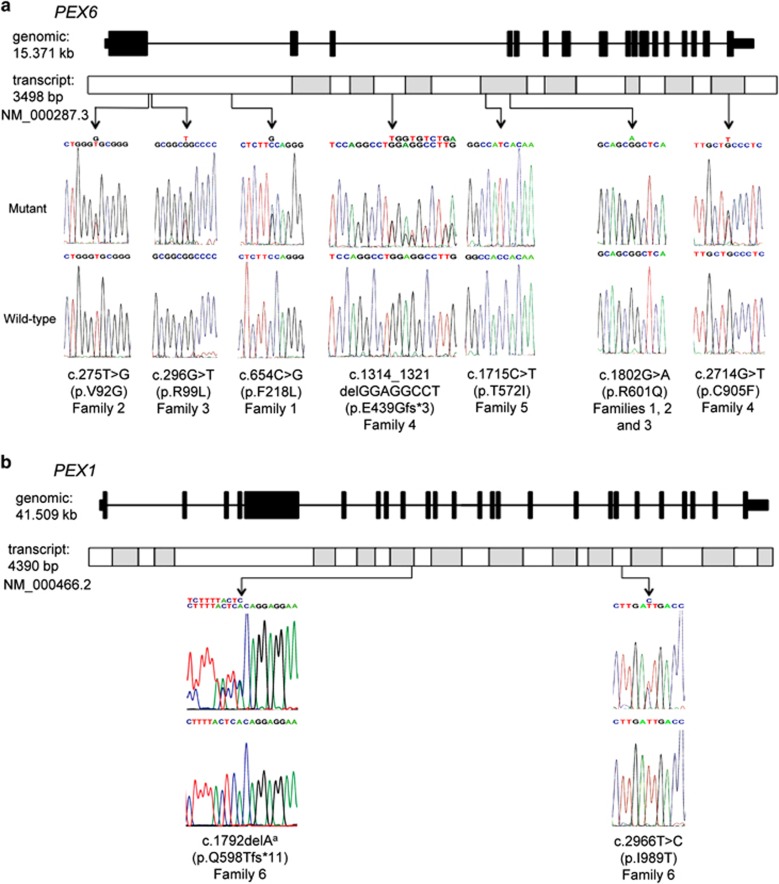
Sanger sequencing and genomic locations of the mutations identified in this study. (**a**) A schematic diagram of *PEX6* genomic structure and transcript shows the location and sequence traces of seven mutations identified in this study. (**b**) A schematic diagram of *PEX1* genomic structure and transcript shows the location and sequence traces of two mutations identified in this study. ^a^The reverse sequence trace is shown for the *PEX1* c.1792delA variant.

The variant files were filtered to remove synonymous, non-coding, intronic and intergenic variants other than those affecting splice donor and acceptor sites. The remaining list was then further filtered to identify biallelic variants. In each family this revealed either *PEX1* or *PEX6* variants that are predicted to be pathogenic by various mutation prediction software packages (Supplementary Table S4) and are rare (MAF<0.01) or absent in human variant databases, including dbSNP142, the Exon Variant Server (EVS; http://evs.gs.washington.edu/EVS/) and the Exome Aggregation Consortium (ExAC; http://exac.broadinstitute.org/) (Table 1 and Figure 2). These included four novel and two known missense variants and an 8 bp deletion in *PEX6*, together accounting for HS in five families, and a known missense and novel frameshift variants in *PEX1* in the remaining family. Previously unreported missense variants, c.654C>G, p.(F218L) and c.2714G>T, p.(C905F), were found to affect residues that are conserved in 12 other mammalian species analysed and in zebrafish (Supplementary Figure S4). Variants c.275T>G, p.(V92G) and c.296G>T, p.(R99L) affect a region of the PEX6 protein that is absent in three mammalian species analysed and is not conserved in zebrafish, but is conserved in the remaining nine mammalian species assessed.

HS patients in three of the families, all from the USA, were compound heterozygotes for the known *PEX6* missense variant c.1802G>A, p.(R601Q) (rs34324426). In each case, this was observed in combination with a novel *PEX6* missense variant; c.[1802G>A][654C>G], p.[(R601Q)][(F218L)] in Family 1, c.[1802G>A][275T>G], p.[(R601Q)][(V92G)] in Family 2 and c.[1802G>A][296G>T], p.[(R601Q)][(R99L)] in Family 3. The c.1802G>A p.(R601Q) variant has been reported previously in seven ZSSD patients^8, 21^ and one HS patient^6^ always in a compound heterozygous state. Ethnicities were not given for the individuals with ZSSD but they were identified in US studies and the HS patient was from the UK, which together with our own findings suggest that this may be a common allele in the US/UK. We therefore carried out haplotype analyses on Families 1, 2 and 3 to determine whether these families carried the allele on a common founder haplotype. By genotyping nearby microsatellite markers and examining the zygosity of known SNPs in WES data from patients, we noted that the c.1802G>A, p.(R601Q) variant is consistently associated with a haplotype of two SNPs and one microsatellite spanning a region of 779 kb (Supplementary Tables S5 and Supplementary Figure S5). This suggests that the c.1802G>A, p.(R601Q) change is a founder variant, though the relatively short range over which the conserved haplotype extends makes this difficult to prove unequivocally, and may imply that it arose many generations ago.

In UK Family 4, a known heterozygous frameshift variant in *PEX6,*^21, 22^ was identified, together with a novel missense variant c.[1314_1321delGGAGGCCT][2714G>T], p.[(E439Gfs*3)][(C905F)]. For Family 5, of Yemenite Jewish Israeli origin and not known to be consanguineous, a known homozygous variant, c.[1715C>T][1715C>T], p.[(T572I)][(T572I)], in *PEX6* was identified.^21, 23^ This variant was previously reported in an individual of mixed Yemenite and Ashkenazi Jewish origin, initially diagnosed with Usher syndrome and subsequently diagnosed with ZS.^23^

In Family 6, of Chinese origin, a novel *PEX1* frameshift variant was identified, in combination with a known missense variant,^24^ c.[1792delA] [2966T>C], p.[(Q598Tfs*11)][(I989T)]. Plasma from Individual II:1 underwent analysis for a variety of peroxisomal parameters (Supplementary Table S8). All results were within the reference range except hexacosanoate (C26:0) concentration, which may have been elevated due to dietary factors or the conditions of sample ascertainment. At the time of writing, no other individual from this study has been tested in this way, although individuals from Families 2 and 3 will undergo such testing in future.

### Pex1, Pex6 and Pex14 immunoreactivity in the adult mouse retina

The pathology associated with the retina of individuals with HS and other ZSSD suggests that peroxisomes are crucial to the development and/or maintenance of a functional retina. Therefore, we utilised immunohistochemical staining to determine the locations of Pex1 and Pex6 within the retina. Pex14 expression was also analysed since it has been shown to be an optimal marker for identification and localisation of peroxisomes in a variety of cell types.^25^

**Figure 3:**
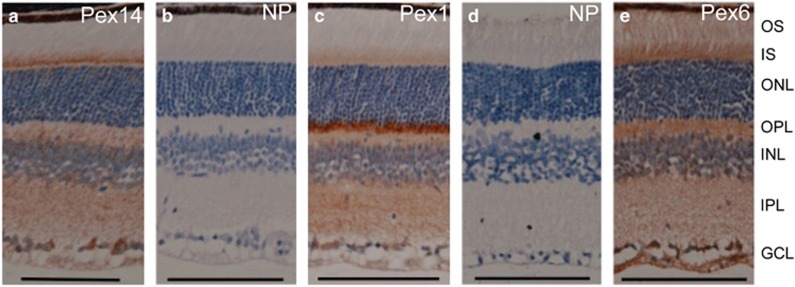
Immunohistochemical staining of murine retina. (**a**) anti-PEX14 antibody followed by labelled polymer-HRP anti-rabbit reagent. (**b**) No primary antibody control: secondary antibody (labelled polymer-HRP anti-rabbit reagent) only. (**c**) anti-PEX1 antibody followed by labelled polymer-HRP anti-rabbit reagent. (**d**) No primary antibody control: control secondary antibody (rabbit anti-goat antibody) only. (**e**) Anti-Pex6 antibody followed by rabbit anti-goat antibody. Scale bars represent 100 μm. Abbreviations: GCL, ganglion cells layer; INL, inner nuclear layer; IPL, inner plexiform layer; IS, inner segment; NP, no primary antibody control; ONL, outer nuclear layer; OPL, outer plexiform layer; OS, outer segment.

Although peroxisomes are known to be ubiquitous cellular organelles, staining of peroxisomal membrane proteins in the retina has only recently been described for Pex6 and PEX6 in murine and human retina, respectively. The study found that Pex6 was detected in nearly all of the layers of the neuronal retina but that staining was most intense in the ciliary region of the photoreceptors and the inner segment.^26^ Therefore, we utilised antibodies raised against PEX1, Pex6 and PEX14 and similarly found staining throughout the retina with the exception of the photoreceptor outer segment in all cases (Figure 3a–e). The ganglion cell layer (GCL) and the photoreceptor inner segment showed the most intense Pex14 immunoreactivity (Figure 3a). For Pex1, the outer plexiform layer (OPL) stained intensely, with strong staining also present in the GCL and the inner plexiform layer (Figure 3c). For Pex6, the most intense staining was present in the GCL and the external limiting membrane (ELM) (Figure 3e). In all cases, retinal sections incubated without the primary antibody but with the same secondary antibodies did not reveal nonspecific staining (Figure 3b and d).

## Discussion

This study confirms the report by Ratbi and co-workers^6^ that HS is caused by variants in *PEX1* and *PEX6* and highlights *PEX6* variants as the more common cause of the HS phenotype. Here we report one further HS family with a combination of a known missense and novel frameshift variant in *PEX1*, together with five HS families in which HS is due to four novel and two known missense variants and an 8 bp deletion in *PEX6*. We also report the detection and distribution of Pex1, Pex6 and Pex14 immunoreactivity within the mouse retina.

Analysis of the 14 *PEX* genes implicated in ZSSD in over 600 patients with the more severe ZD, NALD or IRD has shown that 58% of mutations are in *PEX1*, with *PEX6* accounting for a further 16%, *PEX12* for 9% and the remaining 11 genes each accounting for 4% or less.^9^ By comparison, Ratbi and co-workers^6^ found four HS cases with *PEX1* mutations and two with *PEX6* mutations, mirroring the frequency observed for ZSSD. In this case series however, *PEX6* variants predominate, bringing the total across both studies to seven families/cases with *PEX6* variants and five with *PEX1* variants in HS families. This may imply that variants in *PEX6* have less severe consequences and are therefore found more commonly in the milder HS than in the more severe ZSSD.

Previous studies of ZSSD have reported evidence of genotype–phenotype correlation with respect to mutation type in both *PEX1* and *PEX6*. The more severe cases, those diagnosed as ZS, have more deleterious genotypes, including homozygous stop and frameshift variants, while NALD and IRD cases, at the milder end of the ZSSD spectrum, include missense variants, splicing defects and late truncating stops that may leave a partially functional protein.^15, 16, 17^ Ratbi and co-workers^6^ extended this observation to HS, now shown to be the mildest form of ZSSD, by proving that HS is caused by genotypes that include hypomorphic alleles. The hypomorphic nature of at least one variant in each HS genotype was confirmed using a cDNA transfection complementation assay, including the c.1802G>A, p.(R601Q) variant identified here in Families 1, 2 and 3.^6^ The remaining HS variants identified in this study have not been tested in this way, but each HS genotype documented includes at least one missense variant.

The presence of AI and the absence of abnormal brain findings or impaired liver function represent the phenotypic features that delineate HS from the other ZSSDs. Despite this, there have been reports of IRD patients with AI,^27, 28, 29^ with one suggesting that AI is a common finding in IRD patients.^27^ A recent study described a family with a homozygous missense variant in *PEX6* with a combination of microcephaly, developmental delay, white matter changes, AI as well as sensory defects.^26^ Therefore it is difficult to assign individuals to HS or IRD diagnoses as it is becoming increasingly clear that their phenotypes appear to overlap.

Many of the variants identified in the HS patients reported here are previously unreported in the very substantial previous literature on ZSSD. This may reflect the relatively mild ZSSD phenotype seen in HS patients, which may have led to misdiagnosis as Usher syndrome, or simply the private nature of many mutations in ZSSD.^30^ By contrast, the missense variant c.1802G>A, p.(R601Q) (rs34324426) has been identified in three of the families detailed here and therefore in four out of the seven HS families with *PEX6* mutations reported to date. This variant is reported in ExAC at an allele frequency of 0.003192 (316 alleles out of 98 988), including four homozygotes, and has been found in seven compound heterozygous ZSSD patients with *PEX6* mutations, although no description of the phenotype of these patients is given.^8, 21^ The apparent common origin of many of these cases led us to test the hypothesis that they derive from a common ancestor. Our findings showed that the c.1802G>A, p.(R601Q) variant is consistently associated with a haplotype spanning 778 kb adjacent to *PEX6*, suggesting that this is indeed a founder variant.

The homozygous c.1715C>T, p.(T572I) variant identified in Family 5 has also been reported previously.^21, 23^ In one report, a patient with the same homozygous genotype and same Yemenite Jewish ethnic origin had initially been diagnosed with Usher syndrome.^23^ Biochemical analysis of the patient, undertaken only after their child exhibited an intermediate NALD/ZS phenotype, revealed mild peroxisomal biochemical dysfunction. Interestingly, the patient was not reported to have enamel hypoplasia, a consistent finding in all HS patients so far, so the connection with HS was not made at the time. This may be due to the influence of additional genetic or environmental modifiers, or the tooth abnormality may have been present but assumed to be unconnected at the time of reporting. The c.1715C>T, p.(T572I) variant may therefore represent a founder mutation within the Yemenite Jewish population, and patients from that population diagnosed with Usher syndrome or isolated sensorineural hearing loss should be reevaluated and examined for AI.

Staining of mouse retina showed that peroxisomal membrane proteins are expressed in many compartments of the tissue. A previous study of the mouse neuronal retina detected Pex6 in nearly all of the layers but showed intense staining in the ciliary region and inner segment of the photoreceptors.^26^ The staining pattern seen for Pex6 in this study is similar in that expression was detected throughout the retina, including strong staining of the IS and OPL, although the most intense staining was seen at the GCL and ELM. The differences in the staining pattern between this and the previous study could be due to the use of different detection methods, antibodies or to differences in the age of the mice studied. The staining here for all three Pex proteins suggests that peroxisomes are particularly abundant within the OPL and GCL. Such layers contain features likely to provoke metabolic stress, such as synapses, and therefore may require higher numbers of peroxisomes in order to provide efficient means of detoxification for cell survival. Previous immunohistochemical analysis of developing mouse molar teeth has shown that peroxisomes are present at a high level at the secretory stage of amelogenesis, where they appear in be transported into the Tomes' processes, the structures responsible for the secretion of the enamel matrix.^31^ Pex6 immunoreactivity has also been reported in the Tomes' processes of secretory stage ameloblasts of developing molar teeth. Therefore in HS patients, a reduction in the number or efficient function of peroxisomes may compromise the function or survival of the ameloblasts, ganglion cells and the cells of the OPL.

For the majority of the patients described in this report, the diagnosis was of Usher syndrome and AI. All reports of HS to date suggest that only the secondary dentition is affected by AI. However, a diagnosis of AI, whether syndromic as in HS or occurring in isolation, is often delayed until the appearance of the permanent dentition since it is more difficult to recognise in the primary dentition. In HS, it appears that peroxisomal dysfunction has a more significant effect upon amelogenesis of the secondary dentition compared with the primary dentition. Thus at the time of an Usher syndrome diagnosis, any accompanying AI in the primary dentition may not be recognised. Furthermore, since the treatment of AI is administered by different health professionals, it may not be recognised as an aspect of a syndromic disease. We therefore suggest that paediatric patients presenting with sensorineural hearing loss, either in isolation or in combination with vision defects, should be checked for AI, with particular consideration of the radiographic appearances of unerupted teeth. Where there is a suspicion of AI, this should prompt consideration of the alternative diagnosis of HS. Diagnostic testing should utilise sequencing of *PEX1* and *PEX6* rather than the biochemical analyses traditionally used to confirm a ZSSD diagnosis, since results from patients with HS may still reside within the reference ranges.^6^

In summary, we present six further HS families, five with biallelic *PEX6* variants and one with biallelic *PEX1* variants. We also determine the location of peroxisomal membrane proteins in the mature retina of the mouse. Our study brings the total reported HS genotypes to date to seven with biallelic *PEX6* variants and five with *PEX1* variants, suggesting that *PEX6* variants are more common in HS than in other ZSSD phenotypes. All families segregated at least one missense variant, consistent with the hypothesis that HS results from genotypes that include milder hypomorphic alleles. The clinical overlap of HS with the more common Usher syndrome and lack of peroxisomal abnormalities on plasma screening suggests that HS may be under-diagnosed. Recognition of AI is key for the accurate diagnosis of HS.

## Figures and Tables

**Table 1:** Variants identified in *PEX6* and *PEX1* in individuals with HS

*Family*	*Gene*	*Variant*	*Type of variant (DNA level)*	*Predicted amino acid change*	*CADDv1.3*^a^	*Reference*	*Alleles dbSNP142*^b^	*Alleles EVS*^c^	*AllelesExAC*^d^
1	*PEX6*^e^	c.654C>G	Non-synonymous SNV	p.(F218L)	23.8	None	N/A	N/A	N/A
1, 2 and 3	*PEX6*^e^	c.1802G>A	Non-synonymous SNV	p.(R601Q)	35	Yik *et al.*,^8^ Ebberink *et al.*,^9^ Ratbi *et al.*^6^	rs34324426; 5/5008	42/13006	316/98988(includes 4 homozygotes)
2	*PEX6*^e^	c.275T>G	Non-synonymous SNV	p.(V92G)	22.9	None	N/A	N/A	N/A
3	*PEX6*^e^	c.296G>T	Non-synonymous SNV	p.(R99L)	29.4	None	N/A	N/A	N/A
4	*PEX6*^e^	c.1314_1321delGGAGGCCT	Eight nucleotide deletion	p.(E439Gfs*3)	33	Krause *et al.*,^22^ Ebberink *et al.*^21^	rs267608216; no allele frequency stated	1/12518	4/121224
4	*PEX6*^e^	c.2714G>T	Non- synonymous SNV	p.(C905F)	34	None	N/A	N/A	N/A
5	*PEX6*^e^	c.1715C>T	Non- synonymous SNV	p.(T572I)	23.5	Raas-Rothschild *et al.*,^23^ Ebberink *et al.* ^21^	rs61753224; no allele frequency stated	N/A	N/A
6	*PEX1*^f^	c.1792delA	Single-nucleotide deletion	p.(Q598Tfs*11)	35	None	N/A	N/A	N/A
6	*PEX1*^f^	c.2966T>C	Non- synonymous SNV	p.(I989T)	29.6	Maxwell *et al.*,^24^	rs61750427; 2/10016	N/A	6/120686

For each variant, its CADDv1.3 score (a measure of deleteriousness) is stated and it is indicated whether the variant has been described before, either in a publication or in a database of human variation. If identified, the frequency of the variant in the database studied is stated as the number of reported variant alleles over the total number of alleles sequenced at that locus.

All databases were accessed 7 March 2016.

a Combined Annotation Dependent Depletion (CADD) v1.3 (http://cadd.gs.washington.edu/info).

b Database of Single Nucleotide Polymorphisms build ID: 142 (dbSNP142), Bethesda (MD): National Center for Biotechnology Information, National Library of Medicine (http://www.ncbi.nlm.nih.gov/SNP/).

c Exon Variant Server (EVS) (http://evs.gs.washington.edu/EVS/) version 0.3

d Exome Aggregation Consortium (ExAC), Cambridge, MA (http://exac.broadinstitute.org).

e *PEX6*: Ensembl: ENST00000304611 or GenBank: NM_000287.3.

f *PEX1*: Ensembl: ENST00000248633 or GenBank: NM_000466.2.
