# Supplementary material for: Spectrum of PEX1 and PEX6 variants in Heimler syndrome
Source: Eur J Hum Genet. 2016 Jun 15;24(11):1565–71. doi: 10.1038/ejhg.2016.62 (PMC5026821; doi:10.1038/ejhg.2016.62)
Supplement: Supplementary Information [file ejhg201662x1.docx]

**Supplementary data**

| Gene | Exon | Forward primer (5′-3′) | Reverse primer (5′-3′) | Size (bp) |
| --- | --- | --- | --- | --- |
| *PEX6* | 1(a) | CCTTTTCCTCTGGCCTCC | GAGCTCAGTCACAGCCAGC | 597 |
|  | 1(b) | GTCTTGCGGGTCCTGGAG | GGCTGTGAAGTGTTCGATGA | 698 |
|  | 1(c) | ACTCGGCTGGCTGTGACT | CAAAGTCCGGGATGATATGG | 536 |
|  | 4-5 | TCCATCTGCATTCCTTTTCC | AGGAAGGTCCTCCCAATCC | 585 |
|  | 8 | CCTAGCACCCACCTCACTTC | TCACAAGGCAACAGGACTGA | 387 |
|  | 13 | CATGTATGTGGGCCAAAGTG | TGGACTCTGAAGACTGCTGTG | 483 |
|  | 16 | CATGCAACATGCAGGATGAG | GGTCTCTCTGTGTTGCCCA | 371 |
| *PEX1* | 10 | ACTGGGAAGGCAAAATTCAG | TGGTCAAAACCCAAAGAAAGAAAGAT | 296 |
|  | 19 | ACCTGGCAGAAGTAAAGCTCA | GCTGCTACTAGTCGCCCTGA | 166 |

**Table S1** Primer sequences for Sanger sequencing of *PEX6* and *PEX1* variants identified by WES.

|  | Family 1 | | | Family 2 | Family 3 | Family 4 | Family 5 | | Family 6 |
| --- | --- | --- | --- | --- | --- | --- | --- | --- | --- |
|  | III:1 | III:2 | III:3 | II:1 | II:1 | II:2 | II:1 | II:2 | II:1 |
| Origin | USA | USA | USA | USA | USA | UK | Israel | Israel | UK born  Chinese |
| Sex | M | F | M | M | F | F | F | F | M |
| Age (years) at last assessment | NA | NA | NA | 12 | 7 | 11 | 35 | 22 | 45 |
| Amelogenesis imperfecta | + | + | + | + | + | + | + | + | + |
| Intellect, known psychiatric disorder | NA | NA | NA | N | N | N | Mild LD | Mild LD | Mild LD;  paranoid schizophrenia |
| Skin | NA | NA | NA | Hyper-pigmentation on left arm and shoulder | N | NA | Dryness; light facial lesions | Light facial lesions | Dry split skin on hands; ichthyosis  over limbs |
| SNHL |  |  |  |  |  |  |  |  |  |
| Bilateral or unilateral | B | B | B | B | NA | B | B | B | B |
| Age (years) of diagnosis | NA | NA | NA | 1.5 | 3 | 0 | 0 | 0 | Early childhood |
| Degree of hearing loss | NA | NA | NA | Mo to S | Mo to S | P, some VD | Mo to P | Mi to Mo | S |
| Nail Abnormalities |  |  |  |  |  |  |  |  |  |
| Beau’s lines | NA | NA | NA | + | - | NA | - | - | + |
| Other nail changes | NA | NA | NA | - | - | NA | - | Longitudinal stripes | - |
| Ocular Features |  |  |  |  |  |  |  |  |  |
| Retinal dystrophy | + | + | + | + | + | + | + | + | + |

**Table S2** Clinical details of individuals with HS.

Abbreviations are as follows: B bilateral; F female; LD learning disability; M male; Mi mild; Mo moderate; N normal; NA not ascertained; P profound; S severe; SNHL sensory neural hearing loss; VD vestibular dysfunction.

**
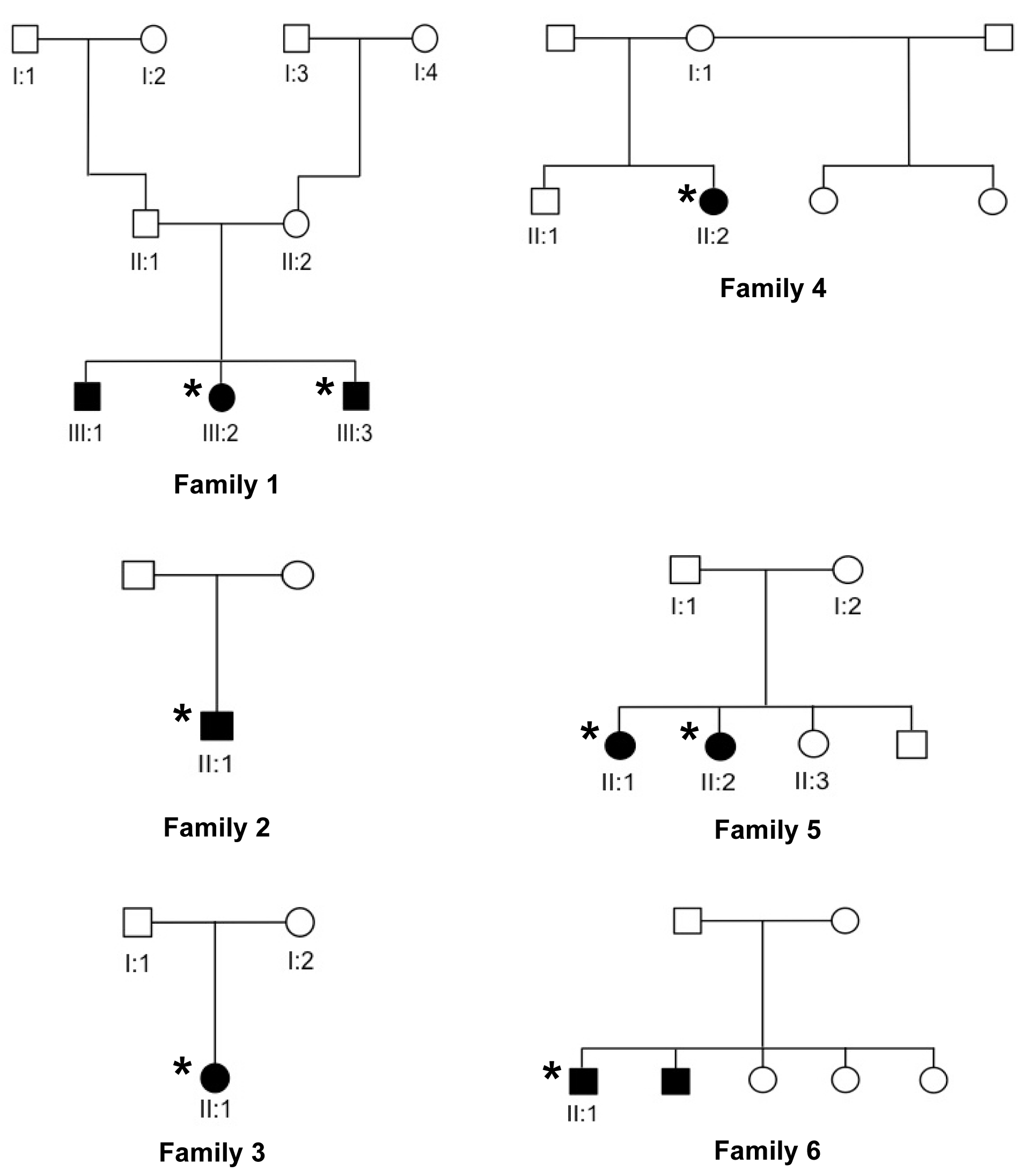
**

**Figure S1** Pedigrees of the families affected by HS. Individuals recruited to the study and in which segregation of identified variants was tested, are denoted by the presence of an identifier on the pedigrees. Asterisks denote individual’s DNA underwent whole exome sequencing.

**
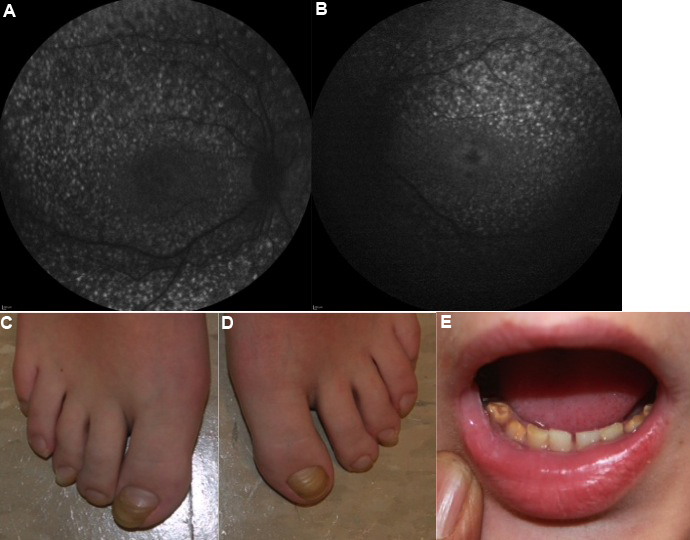
**

**Figure S2** Clinical images of Family 2 II:1. A and B: Fundus autofluorescence of right (A) and left eye (B). C and D: Photographs of II:1’s right (C) and left (D) feet show toenails with Beau’s lines that are most apparent on the halluces. E: Photograph of part of the lower dentition. AI is most apparent in the canine and molar teeth.


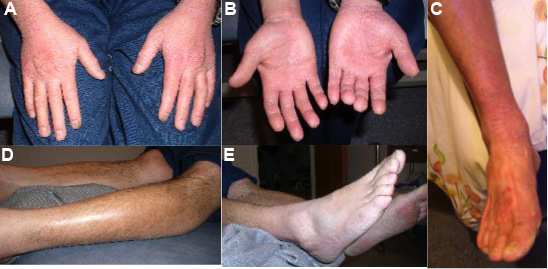


**
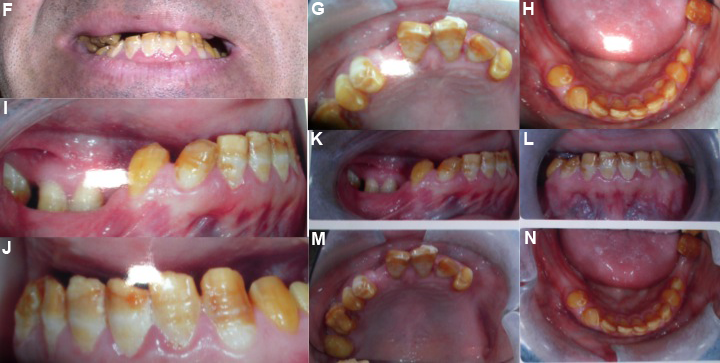
**

**Figure S3** Clinical Images of Family 6 II:1. Photographs of individual’s hands (A and B) that have dry, split skin and right leg (C), legs (D) and feet (E) that display ichthyosis. Photographs of II:1’s dentition: Thirteen teeth have been extracted and those that remain display AI (F to N).

| Family | Sample | Capture reagent | Depth of coverage | % bases covered by >4 reads | % bases covered by >9 reads | % bases covered by >14 reads | % bases covered by >19 reads | % bases covered by >24 reads | % bases covered by >29 reads |
| --- | --- | --- | --- | --- | --- | --- | --- | --- | --- |
| 1 | III:2 | SSV4 | 89.76 | 99.4 | 98 | 95.9 | 92.9 | 89.4 | 85.4 |
| 1 | III:3 | SSV5 | 37.84 | 98 | 93.9 | 86.8 | 77.9 | 68.3 | 58.8 |
| 2 | II:1 | SSV5 | 58.23 | 98.9 | 97.5 | 94.8 | 90.5 | 84.9 | 78.5 |
| 3 | II:1 | SSV5 | 83.23 | 98.9 | 98 | 96.7 | 94.8 | 92.2 | 89 |
| 4 | II:2 | SSV5 | 65.44 | 98.8 | 97.5 | 95.5 | 92.3 | 88.2 | 83.2 |
| 5 | II:1 | SSV5 | 64.68 | 98.6 | 97.3 | 95.1 | 91.9 | 87.5 | 82.4 |
| 5 | II:2 | SSV5 | 76.82 | 99 | 98.3 | 97.1 | 95.3 | 92.6 | 89 |
| 6 | II:1 | SSV5 | 67.92 | 98.5 | 96.9 | 94.6 | 91.3 | 87.2 | 82.3 |

**Table S3** Alignment statistics for whole exome sequencing. Alignment statistics were generated using the regions targetted by the relevant capture reagent: SSV4 - SureSelectXT Human All Exon V4 or SSV5 - SureSelectXT Human All Exon V5.

| **Gene** | **Variant** | **Predicted Amino Acid Change** | **SIFT^c^** | **Polyphen2^d^** | **Mutation Taster^e^** | **CADD v1.3^f^** | **Grantham Score^g^** |
| --- | --- | --- | --- | --- | --- | --- | --- |
| *PEX6*^a^ | c.275T>G | p.(V92G) | Damaging,  0 | Probably damaging,  0.987 | Disease causing,  1 | 22.9 | 109 |
| *PEX6*^a^ | c.296G>T | p.(R99L) | Damaging,  0 | Probably damaging, 0.982 | Disease causing,  0.787 | 29.4 | 102 |
| *PEX6*^a^ | c.654C>G | p.(F218L) | Damaging, 0.005 | Possibly damaging,  0.755 | Disease causing,  0.982 | 23.8 | 22 |
| *PEX6*^a^ | c.1314_1321  delGGAGGCCT | p.(E439Gfs*3) | N/A | N/A | Disease causing,  1 | 33 | N/A |
| *PEX6*^a^ | c.1715C>T | p.(T572I) | Damaging, 0.001 | Probably damaging,  0.963 | Disease causing,  1 | 23.5 | 89 |
| *PEX6*^a^ | c.1802G>A | p.(R601Q) | Damaging, 0.001 | Probably damaging, 0.964 | Disease causing,  1 | 35 | 43 |
| *PEX6*^a^ | c.2714G>T | p.(C905F) | Damaging, 0.01 | Probably damaging, 0.961 | Disease causing,  1 | 34 | 205 |
| *PEX1*^b^ | c.1792delA | p.(Q598Tfs*11) | N/A | N/A | Disease causing,  1 | 35 | N/A |
| *PEX1*^b^ | c.2966T>C | p.(I989T) | Damaging, 0.001 | Probably damaging, 0.995 | Disease causing,  1 | 29.6 | 89 |

**Table S4** Summary of bioinformatics analyses undertaken to predict the pathogenic nature of the variants identified in *PEX6* and *PEX1* in individuals with HS.

For each variant, its predicted pathogenic effect and conservation are calculated using a variety of pathogenicity prediction and conservation score software. SIFT and Mutation Taster annotations were based on the relevant Ensembl transcript, Polyphen-2 annotations were based on the relevant RefSeq protein.

^a^ *PEX6*: Ensembl transcript: ENST00000304611 or PEX6: RefSeq protein NP_000287.

^b^ *PEX1*: Ensembl transcript: ENST00000248633 or PEX1: RefSeq protein NP_000457.

^c^ SIFT, http://sift.jcvi.org/ [Ng, P.C. et al. (2003). Nucleic Acids Res. 31, 3812-4];

^d^ PolyPhen2, http://genetics.bwh.harvard.edu/pph2/ [Adzhubei, I.A. et al. (2010). Nat. Methods 7, 248-9];

^e^ Mutationtaster, http://www.mutationtaster.org/ [Schwarz, J.M. et al. (2010). Nat. Methods 7, 575-6];

^f^ Combined Annotation Dependent Depletion (CADD) v1.3, <http://cadd.gs.washington.edu/info> [Kircher, M. et al. (2014) Nat. Genet. 46, 310-5];

^g^ Grantham Score [Grantham, R. (1974) Science 185, 862-4]

**A**

p.V92G p.R99L

🡻 🡻

Human 82 RLLALGSGAWVRARAVRRPPALGWALLG 109

Chimpanzee 82 RLLALGSGAWVRARAVRRPPALGWALLG 109

Marmoset 82 RLLALGSGAWVRARPVRRPPALGWALLG 109

Guinea pig 82 QLLALGSGAQVRARPVRRPPALGWALLG 109

Mouse 82 RVLALGPGARVRARLVRRPPALGWALLA 109

Rat 82 RVLALSPGARVRARPVRRPPALGWALLG 109

Elephant 82 RLLALDSGAWVRARPVRRPPALGWALLG 109

Dog 82 RLLALGSGAWVRARPVRRPPALGWALLG 109

Cow 82 QLLALGSGAWVRARPVRRPPSLGWALLG 109

Sheep 82 QLVALGSGAWVRARPVRRPPSLGWALLG 109

Cat 82 RLLAL----------------------- 86

Sperm whale 37 -----------------------WALLG 41

Horse 0 ---------------------------- 0

Zebrafish 89 KHYGLREQSWGTLRPQSLLPLKKIVIGA 116

**B**

p.F218L

🡻

Human 208 SRSCLRGLGLFQGEWVWVAQA 228

Chimpanzee 208 SRSCLRGLGLFQGEWVWVARA 228

Marmoset 208 SRRCLRGLGLFQGEWVWVARA 228

Guinea pig 210 SRTCLRGLGLFQGEWVWVARA 230

Mouse 208 SRSCLRSLGLFQGEWVWVAQV 228

Rat 208 SRSCLRSLGLFQGEWVWVARV 228

Elephant 207 SRRCLRGIGLFQGEWVWVARA 227

Dog 208 SRSCLRSLGLFQGEWVWVTRA 228

Cow 208 SRSCLRSLSLFQGEWVWVTRA 228

Sheep 190 SRSCLRSLGLFQGEWVWVTRA 210

Cat 172 SRSCLRSLGLFQGEWVWVTRA 192

Sperm whale 140 SRSCLRSLSLFQGEWVWVTRA 160

Horse 57 SRSCLRSLGLFQGEWVWVTRA 77

Zebrafish 287 SRSLLVKLGVFNGEWVIASVP 307

**C**

p.C905F

🡻

Human 895 SVSLVNVLDCCPPQLTGADLY 915

Chimpanzee 895 SVSLVNVLDCCPPQLTGADLY 915

Marmoset 895 SVNLVNVLDCCPPQLTGADLY 915

Guinea pig 897 SVSLVNVLDHCPPQLTGADLY 917

Mouse 896 SVSLANVLDCCPPQLTGADLY 916

Rat 893 SVSLMNVLDCCPPQLTGADLY 913

Elephant 893 TVSLVRVLDHCPPQLTGADLY 913

Dog 895 SVSLVNVLDRCPPQLTGADLY 915

Cow 895 SVSLVDVLDHCPPQLTGADLY 915

Sheep 879 SVSLVDVLDHCPPQLTGADLY 899

Cat 859 SVSLVNVLDRCPPQLTGADLY 879

Sperm whale 827 SVSLVDVLDHCPPQLTGADLY 847

Horse 745 SVSLVNVLDRCPPQLTGADLY 765

Zebrafish 986 SVCLSDIVESCPPRLTGADLY 1006

**Figure S4** Clustal Omega multiple sequence alignment of homologous protein sequences for the sequences surrounding each previously unpublished missense variant identified in HS individuals; A: p.V92G and p.R99L; B: F218L; C: p.C905F. Shaded residues indicate those that are identical to the human residue at the same position.

PEX6 sequences used: Human, *Homo sapiens* NP_000278.3; Chimpanzee, *Pan troglodytes* XP_003950894.1; Marmoset, *Callithrix jacchus* XP_002746596.1; Guinea pig, *Cavia porcellus* XP_003473901.1, Mouse, *Mus musculus* NP_663463.1; Rat, *Rattus norvegicus* NP_476466.1; Elephant, *Loxodonta africana* XP_003403954.1; Dog, *Canis lupus familiaris* XP_538926.2; Cow, *Bos taurus* NP_001179876.1; Sheep, Ovis aries XP_004018878.1; Cat, *Felis Catus* XP_011280631.1; Sperm whale, *Physeter catodon* XP_007107679.1; Horse, *Equus caballus* XP_005614975.1; Zebrafish, *Danio rerio* XP_009294800.1

| Marker | Physical location (hg19 / GRCh37) | Genetic location (Genethon) | Genetic location (Marshfield) | Genetic location (Decode) | Forward primer (5′-3′) | Reverse primer (5′-3′) | Size (bp) |
| --- | --- | --- | --- | --- | --- | --- | --- |
| D6S1552  AFM157XE11 | 41955110-41955373 | 63.50 | 63.28 | N/A | AGCCTGAACGACAGAACAAG | CTGCTTAACTTNAGATCTTTGGTAT | 156-184 |
| D6S400  UT2081 | 42156381-42156758 | N/A | 63.28 | N/A | AGCCTGGGTAACTTAGTGAG | TCTTTGCTCAAGTCTTTCTCC | 182 |
| D6S1582  AFMA272ZB5 | 43098617-43098961 | 65.20 | 65.14 | N/A | CTAGGTAGTCAGGTGGTCATAGTC | AGTAGGGCTGGAACCTTCT | 133-151 |
| D6S282  AFM184XA11 | 43234575-43234945 | 66.40 | 66.37 | 66.10 | ATGGCCCAGACAGTGGGTAT | ATGGTTTGTGCAGGTTCAGA | 108-126 |
| D6S271  AFM136YF8 | 43500818-43501193 | 66.40 | 66.37 | 66.44 | AACAATTGGGAAATGGCTTA | TAGGTTGTGGTGGGTGTTAC | 166-208 |
| D6S1604  AFMB007XA9 | 43679700-43680083 | 66.40 | 66.37 | N/A | CTGGGACTACAGGCATGAGC | CTAGGACTGGGCAGGATTTG | 189-205 |
| D6S1650  AFMB334YG9 | 44130031-44130426 | 68.70 | 68.65 | N/A | GGGCTCCACTGTTTAACC | CCCTCGGGCTATGATTAC | 113-126 |

**Table S5** Summary of Sequence Tagged Site (STS) markers genotyped to analyse the haplotype surrounding the shared *PEX6* c.1802G>A variant identified in HS families 1, 2 and 3.

| dbSNP build 142 | Physical location (hg19 / GRCh37) | Reference (allele frequency) | Alternative (allele frequency) | Forward Primer (5′-3′) | Reverse Primer (5′-3′) |
| --- | --- | --- | --- | --- | --- |
| rs7770760 | 42915403 | C (52.935%) | T (47.064%) | TCCTGGGTTCACGCCATT | TGCTAGCAGATCCACCCAC |
| rs9471978 | 42929533 | C (50.319%) | T (49.681%) | GATGAAGTCGTGCTCTGTCG | CCATTTGAGAGACAGACAAGCT |
| rs34324426 | 42935188 | C (99.900%) | T (0.100%) | CCTAGCACCCACCTCACTTC | TCACAAGGCAACAGGACTGA |

**Table S6** Summary of Single Nucleotide Polymorphisms sequenced to analyse the haplotype surrounding the shared *PEX6* c.1802G>A variant (rs34324426) identified in HS families 1, 2 and 3. Allele frequencies relate to dbSNP build 142.


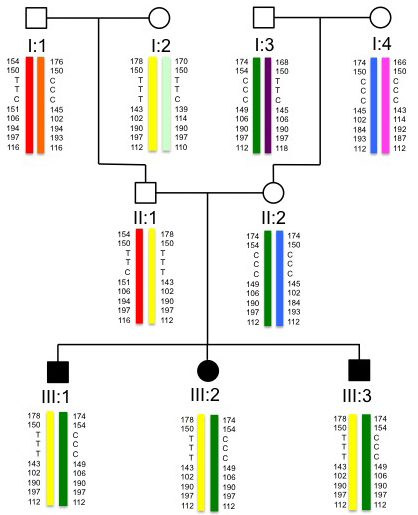


Family 1


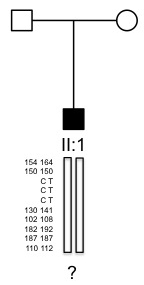
 **
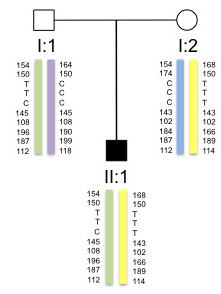
**

Family 2 Family 3

**Figure S5** Analysis of the haplotype of the region surrounding the *PEX6* c.1802G>A variant for families 1, 2 and 3. Results are shown for 8 microsatellite markers and 3 SNPs, including the *PEX6* c.1802G>A. Markers used are described in Table S5 and a summary of the results can be found in Table S6. Haplotypes are represented by coloured bars (each colour represents a different haplotype) The numbers alongside each haplotype bar correspond to the size of each allele amplified by the microsatellite markers, in base pairs. The markers are displayed in order of their physical location.

| **Marker** | **Physical location (hg19 / GRCh37)** | **Genetic location (Marshfield)** | **Result - Family 1** | **Result - Family 2** | **Result - Family 3** |
| --- | --- | --- | --- | --- | --- |
| D6S1552  AFM157XE11 | 41537757-41538086 | 62.28 | 178 | 154 / 164 | 168 |
| D6S400  UT2081 | 41955110-41955373 | 63.28 | 150 | 150 / 150 | 150 |
| rs7770760 | 42915403 | N/A | T | C / T | T |
| rs9471978 | 42929533 | N/A | T | C / T | T |
| rs34324426 | 42935188 | N/A | T | C / T | T |
| D6S1582  AFMA272ZB5 | 43098617-43098961 | 65.14 | 143 | 130 / 141 | 143 |
| D6S282  AFM184XA11 | 43234575-43234945 | 66.37 | 102 | 102 / 108 | 102 |
| D6S271  AFM136YF8 | 43500818-43501193 | 66.37 | 190 | 182 / 192 | 166 |
| D6S1604  AFMB007XA9 | 43679700-43680083 | 66.37 | 197 | 187 / 187 | 189 |
| D6S1650  AFMB334YG9 | 44130031-44130426 | 68.65 | 112 | 110 / 112 | 114 |

**Table S7** Summary of genotyping results to analyse the haplotype surrounding the shared *PEX6* c.1802G>A variant (rs34324426) identified in HS families 1, 2 and 3. Shading indicates a region potentially shared between all 3 families.

| **Peroxisomal parameter (Plasma)** | | **Family 6. II:1** | **Reference range** |
| --- | --- | --- | --- |
| VLCFA  concentration (μmol/l) | Docosanoate C22:0 | 75.0 | 15.0-112.0 |
|  | Tetracosanoate C24:0 | 68.0 | 14.0-80.0 |
|  | Hexacosanoate C26:0 | 2.15^a^ | 0.33-1.50 |
| VLCFA ratio | C24:C22 | 0.910 | 0.440-0.970 |
|  | C26:C22 | 0.029 | 0.005-0.030 |
| Phytanate (μmol/l) |  | 3.6 | 0.0-19.3 |
| Pristanate (μmol/l) |  | 0.22 | 0.00-1.88 |

**Table S8** Peroxisomal parameters in blood of Family 6, Individual II:1.

Sample is from a male, aged 45. Reference ranges indicate the normal range according to the lab in which the assays were performed. Abbreviation is as follows: VLCFA very long chain fatty acids.

^a^ C26 level is above the reference range, however, C26:C22 ratio is within the reference range, therefore this may be due to a dietary influence or the use of a non-fasting sample.
